# Simulation Design of Surface Acoustic Wave Sensor Based on Langasite Coplanar Integration with Multiple Parameters

**DOI:** 10.3390/mi13050705

**Published:** 2022-04-29

**Authors:** Xiaorui Liang, Yongwei Zhang, Fangmeng Xu, Qiulin Tan, Juan Zhang

**Affiliations:** State Key Laboratory of Dynamic Measurement Technology, North University of China, Taiyuan 030051, China; b1906058@st.nuc.edu.cn (X.L.); s1706011@st.nuc.edu.cn (Y.Z.); s1806154@st.nuc.edu.cn (F.X.); b1806011@st.nuc.edu.cn (J.Z.)

**Keywords:** surface acoustic wave, Langasite, multi-parameter, coplanar integration

## Abstract

In the harsh environment of high temperature and high rotation, a single parameter is difficult to satisfy the multi-parameter test requirements of aerospace metallurgy. Therefore, a multi-parameter coplanar integrated surface acoustic wave (SAW) sensor based on Langasite (LGS) is proposed. In this paper, the optimal cut for different measurement parameters is analyzed, and the optimal cut to temperature, pressure and vibration are obtained. The simulation results show that (0°, 138.5°, 25°) LGS has superior second-order temperature sensitivity, the edge of the rectangular sealed cavity is more suitable for pressure sensors, and the optimal cut is (0°, 138.5°, 30°). The stress of the vibration sensor cantilever beam is mainly concentrated on the edge of the fixed end, and the optimal cut is (0°, 138.5°, 35°). Based on the optimal sensitive tangential direction of each sensitive element and the symmetry of the Langasite wafer, the reasonable layout of the coplanar integrated structure with the three parameters of temperature, pressure and vibration is determined. Moreover, according to the optimal orientation selection and reasonable structure layout of each parameter, combined with frequency separation rules, the parameters of interdigital electrode were determined, and the idea of multi-parameter integrated design was simulated and verified.

## 1. Introduction

Surface acoustic wave (SAW) devices have attracted increasing attention in the field of aerospace, metallurgy, fuel system health monitoring and other applications in the harsh environment such as high temperature and high pressure for their advantages of wireless communication and passive power supply [1,2,3,4,5]. At present, a single parameter device cannot satisfy the measurement requirements of in situ real-time testing of critical components in a composite environment, and multi-functional integrated sensors have become the trend of development in the future.

A LiNbO_3_/SiO_2_/Si structure is adopted to manufacture a SAW temperature sensor, which has good linearity and resolution at 20–100 °C [6]. Another temperature/pressure combined sensor using LiNbO_3_ as the base material can measure pressure at 0–6 bar and a temperature of 30–150 °C [7]. Temperature-compensated vibration sensors made of quartz cantilever can measure vibration at a temperature of 25–115 °C [7]. The temperature and pressure dual parameter SAW sensor developed by the Segura-Quijano research group of Columbia Andes University adopts the MEMS technology to manufacture temperature and pressure surface acoustic wave sensors that are based on LiNbO_3_ with a thickness of 0.5 mm. The temperature test range of the sensor is 20 °C to 200 °C, and the pressure test range is 0 to 350 kPa [8]. Its temperature and pressure sensitivity reached 87.81 ppm/C and 0.9 ppm/kPa, respectively. The Romanian Scientific Research Center has developed a temperature and pressure dual-parameter SAW sensor based on the GaN film, which is 1.2 μm thick [9]. The sensor adopts a symmetrical structure with a resonant frequency > 11 GHz, so it has high sensitivity. In order to obtain the resonant frequency and bandwidth distribution of resonators, the North University of China established a model of resonant SAW multi-parameter integrated sensors by using COMSOL Multiphysics simulation software. According to the simulation results, a temperature/pressure/humidity three parameter integrated SAW sensor based on LiNbO_3_ was developed, which can work stably in the composite environment of 25–200 °C, 0–42 kPa, 10–90% RH [10]. However, these sensors are generally unusable in high temperature environments due to the degradation or deactivation of crystalline materials in high temperature environments [11]. In order to solve this problem, we used some high-temperature-resistant piezoelectric crystal materials such as LGS. Langasite (LGS) is considered as a promising alternative to achieve multi-parameter integrated sensors in harsh environments such as high temperature. Compared with other materials, Langasite has a high melting point (1470 °C), no phase transition from room temperature to melting point, low sound velocity, zero temperature tangential and good high temperature stability [12,13,14,15].

Although these sensors exhibit excellent performance and multi-parameter integration for stable operation, the current devices cannot achieve the purpose of simultaneous multi-parameter measurement in harsh environments such as high temperatures. To date, sensors based on LGS three-parameter integration for composite harsh environments such as high temperature and high pressure have not been reported. In this paper, we demonstrate a multi-parameter coplanar integration sensor based on Langasite for multi-parameter simultaneous measurement in harsh environments. The sensor is used to measure a variety of parameters by setting corresponding sensitive elements for it.

In the cause of improving the performance of SAW sensors manufactured using LGS and the protection of sensitive structures, the material characteristics and simulation of LGS are necessary before the sensor is manufactured. Lu Wenke [16] proposed to use a finite element to simulate SAW stress and strain based on a micro-force sensor, calculate the precise mechanical position of the fork finger sensor (IDT), and study the theoretical research of pressure sensors of different SAW sizes. Onur Tigli et al. [17] introduced finite element modeling (FEM) and performance analysis of SAW devices developed using complementary metal oxide semiconductor (CMOS) technology. A detailed 3D model with 18 CMOS layers and a structured finite element analysis method were designed to extract the acoustic behavior of the substrate and the ZnO piezoelectric material of interest. Bowei Xie et al. [18] studied the dependence of microwave impedance characteristics on the thickness of the piezoelectric layer and metal electrode layer by theoretical analysis and finite element method (FEM), calculated the distribution of electric potential and particle displacement under resonance condition, and analyzed the frequency response. How the thickness of the piezoelectric layer and the electrode affects the frequency response was then systematically evaluated. At present, there are only finite element simulation analyses on single conditions such as force and impedance characteristics to study the coupling relationship between sensitive elements with multiple parameters. In this paper, a simulation analysis of the phase velocity and temperature characteristics of (0°, 138.5°, φ°) LGS is provided for sensors’ design reference, since this tangential LGS crystal has excellent properties [19]. COMSOL Multiphysics was used to simulate the structural changes to optimize the performance of sensors. The position and strain of the cylindrical and rectangular sealed cavities are analyzed to determine the optimal position and tangential direction of the sensor. The displacement and stress of the cavity are also analyzed to avoid damage to the cavity structure. For the vibration sensor, we analyzed the strain and mode of the cantilever beam to improve the performance of the sensor while avoiding the damage to the cantilever beam by resonance. Through the analysis of single-parameter sensor simulation, the optimal sensitive tangent is obtained. According to the symmetry of the LGS crystal, the reasonable layout of the multi-parameter coplanar integration is determined. Due to the multi parameter coplanar integration, the crosstalk easily occurs between the corresponding sensitive frequencies in the full range of each parameter, and the design method of determining that there is no intersection in the sensitive frequency range of each range is proposed. According to the above multi parameter design rules, the multi parameter integrated sensor is simulated and analyzed to achieve three independent resonant frequencies. Multi-parameter coplanar integration design provides a new idea and theoretical basis for the multi-parameter simultaneous measurement of multiple parameters in harsh environments such as aerospace, metallurgy, and fuel system health monitoring under high temperature and high rotation.

## 2. Calculation Principle and Data Preparation

The performance of SAW resonators is mainly affected by the properties of the substrate material, sensitive structure, and the direction of propagation of the sensors. In order to optimize the performance of pressure and vibration sensors in a high temperature environment, COMSOL Multiphysics was used to simulate the sensitive structure and analyze the properties of LGS crystal in this chapter. (0°, 138.5°, φ°) LGS crystals discussed are described using the Euler angle coordinate system. The range of φ varies from 0–90° based on the symmetry of crystal [20]. 

The resonant frequency of an SAW resonator is defined in Equation (1), where f is the resonant frequency of the sensor and λ is the period of the interdigital transducers (IDTs). The shift in the resonant frequency of an SAW device is mainly affected by the phase velocity of the substrate and the period of the (IDTs) of the SAW resonator. The phase velocity of the substrate is susceptible to temperature changes, mechanical disturbances, mass loading, etc., as shown in Equation (2). This facilitates the measurement of physical parameters, such as temperature, pressure, and acceleration [21].

(1) $$f=\frac{v}{\lambda }$$

(2) $$\frac{\Delta v}{v_{0}}=-k_{m}\frac{\Delta m}{m_{0}}+k_{c}\frac{\Delta c}{c_{0}}+k_{\sigma }\frac{\Delta \sigma }{\sigma_{0}}+k_{\varepsilon }\frac{\Delta \varepsilon }{\varepsilon_{0}}-k_{T}\frac{\Delta T}{T_{0}}+\ldots$$

where v is the phase velocity of the substrate, λ is the period of the IDTs, and $k_{i}$ is the sensitivity to each physical parameter. m is the quality, c is the elastic coefficient, σ is the film conductivity, α is the dielectric constant, and T is the temperature.

When the external temperature changes, the material properties of the base material change due to the change of thermal stress, which causes the distance between the IDT and the reflector to change, which affects the propagation speed of surface acoustic waves. The sensing characteristics of the resonant surface acoustic wave temperature sensor expressed by resonant frequency and the shift of resonant frequency can characterize temperature changes.

Piezoelectric materials are used as a substrate in SAW devices to excite and detect surface acoustic waves. Based on the piezoelectric constitutive equation, Newton’s second law (mechanical behavior), and Maxwell’s equation (electrical behavior), the mathematical models of SAW sensors are established [22,23].

The piezoelectric constitutive relationship is [24]

(3) $$\left\{ \begin{array}{l} T_{ij}=C_{{}_{ijkl}}^{E}\cdot S_{kl}-e_{{}_{ijk}}^{T}\cdot E_{k}\\ D_{i}=e_{ikl}\cdot S_{kl}-\varepsilon_{{}_{ij}}^{S}\cdot E_{j} \end{array}\right.$$

where $T_{ij}$ represents the stress tensor, $S_{kl}$ represents the strain tensor of the medium, $D_{i}$ is the electrical displacement (C/m^2^), $E_{k}$ represents the electric field strength (V/m), $C_{{}_{ijkl}}^{E}$ represents the elastic stiffness tensor of the medium (N/m^2^), $e_{{}_{ijk}}^{T}$ represents the piezoelectric tensor (C/m^2^), and $\varepsilon_{{}_{ij}}^{s}$ represents the dielectric tensor (F/m).

The mechanical properties of the material are controlled by Newton’s second law [25]

(4) $$\rho \frac{\partial^{2}u_{i}}{\partial t^{2}}=\sum_{j}\frac{\partial T_{ij}}{\partial x_{j}}$$

where ρ is the density, $x_{i}$ is the global direction and $u_{i}$ is the global displacement.

The electrical properties of the material are controlled by Maxwell’s equations. Since the surface acoustic wave is much slower than the electromagnetic wave, the quasi-static approximation established.

(5) $$E_{i}=-\frac{\partial \varphi }{\partial x_{i}}$$

(6) $$\frac{\partial D_{i}}{\partial x_{i}}=0$$

where φ is the electrical potential.

According to Equations (3)–(6), the wave equation can be established as

(7) $$\sum_{ijk}c_{{}_{ijkl}}^{E}\frac{\partial^{2}u_{l}}{\partial x_{j}\partial x_{k}}+\sum_{jk}e_{kij}\frac{\partial^{2}\varphi }{\partial x_{j}\partial x_{k}}=\rho \frac{\partial^{2}u_{i}}{\partial t^{2}}$$

(8) $$\sum_{kl}e_{jkl}\frac{\partial^{2}u_{l}}{\partial x_{j}\partial x_{k}}-\sum_{jk}\varepsilon_{{}_{jk}}^{s}\frac{\partial^{2}\varphi }{\partial x_{j}\partial x_{k}}=0$$

for *i*, *j*, *k*, *l* = 1, 2 and 3.

Using the above equations, a finite element model of a piezoelectric structure can be established to extract the stress $\sigma_{x}$, strain (longitudinal and transverse: $\varepsilon_{x}$ and $\varepsilon_{y}$), and displacement of the position where sensors is located. 

The constitutive relation for a thin transversely anisotropic piezoelectric material plate polarized in the z-direction can be written as [26]

(9) $$\left\{\begin{array}{c} T_{xx}\\ T_{yy}\\ T_{zz}\\ T_{xy}\\ T_{xz}\\ T_{yz} \end{array}\right\}=\left[\begin{array}{cccccc} {\bar{c}}_{11} & {\bar{c}}_{12} & {\bar{c}}_{13} & {\bar{c}}_{14} & 0 & 0\\ {\bar{c}}_{12} & {\bar{c}}_{11} & {\bar{c}}_{13} & -{\bar{c}}_{14} & 0 & 0\\ {\bar{c}}_{13} & {\bar{c}}_{13} & {\bar{c}}_{33} & 0 & 0 & 0\\ {\bar{c}}_{14} & -{\bar{c}}_{14} & 0 & {\bar{c}}_{44} & 0 & 0\\ 0 & 0 & 0 & 0 & {\bar{c}}_{44} & {\bar{c}}_{14}\\ 0 & 0 & 0 & 0 & {\bar{c}}_{14} & {\bar{c}}_{66} \end{array}\right]\left\{\begin{array}{c} S_{xx}\\ S_{yy}\\ S_{zz}\\ S_{xy}\\ S_{xz}\\ S_{yz} \end{array}\right\}-\left[\begin{array}{cccccc} e_{11} & -e_{11} & 0 & e_{14} & 0 & 0\\ 0 & 0 & 0 & 0 & -e_{14} & -2*e_{11}\\ 0 & 0 & 0 & 0 & 0 & 0 \end{array}\right]\left\{\begin{array}{c} E_{x}\\ E_{y}\\ E_{z} \end{array}\right\}$$

(10) $$\left\{\begin{array}{c} D_{x}\\ D_{y}\\ D_{z} \end{array}\right\}=\left[\begin{array}{cccccc} e_{11} & -e_{11} & 0 & e_{14} & 0 & 0\\ 0 & 0 & 0 & 0 & -e_{14} & -e_{11}\\ 0 & 0 & 0 & 0 & 0 & 0 \end{array}\right]\left\{\begin{array}{c} S_{xx}\\ S_{yy}\\ S_{zz}\\ S_{xy}\\ S_{xz}\\ S_{yz} \end{array}\right\}-\left[\begin{array}{ccc} \varepsilon_{11} & 0 & 0\\ 0 & \varepsilon_{11} & 0\\ 0 & 0 & \varepsilon_{33} \end{array}\right]\left\{\begin{array}{c} E_{x}\\ E_{y}\\ E_{z} \end{array}\right\}$$

LGS is an anisotropic material. When a temperature field is applied, LGS material will undergo thermal change and related physical properties will change, such as the elastic constant, piezoelectric constant, dielectric constant, density, thermal expansion coefficient, etc., which will be affected by temperature. Formula (11) describes the relationship between physical properties and temperature [27]

(11) $$\begin{array}{l} C_{ijkl}(T)=C_{ijkl}(T_{0})[1+\theta_{1}^{C}(T-T_{0})+\theta_{2}^{C}{\left(T-T_{0}\right)}^{2}+\ldots ]\\ e_{kij}(T)=e_{kij}(T_{0})[1+\theta_{1}^{e}(T-T_{0})+\theta_{2}^{e}{\left(T-T_{0}\right)}^{2}+\ldots ]\\ \varepsilon_{kj}(T)=\varepsilon_{kj}(T_{0})[1+\theta_{1}^{\varepsilon }(T-T_{0})+\theta_{2}^{\varepsilon }{\left(T-T_{0}\right)}^{2}+\ldots ]\\ \rho (T)=\rho (T_{0})[1+\theta_{1}^{\rho }(T-T_{0})+\theta_{2}^{\rho }{\left(T-T_{0}\right)}^{2}+\ldots ]\\ \alpha i(T)=\alpha i(T_{0})[1+\theta_{1}^{\alpha i}(T-T_{0})+\theta_{2}^{\varepsilon }{\left(T-T_{0}\right)}^{2}+\ldots ] \end{array}$$

where,

(12) $$\theta_{1}^{C}=\frac{1}{C_{ijkl}}\frac{dC_{ijkl}}{dT},\theta_{2}^{C}=\frac{1}{2}\frac{1}{C_{ijkl}}\frac{d^{2}C_{ijkl}}{dT^{2}},\theta_{1}^{e}=\frac{1}{e_{kij}}\frac{de_{kij}}{dT},\theta_{2}^{e}=\frac{1}{2}\frac{1}{e_{kij}}\frac{d^{2}e_{kij}}{dT^{2}}$$

(13) $$\begin{array}{l} \theta_{1}^{\varepsilon }=\frac{1}{\varepsilon_{kj}}\frac{d\varepsilon_{kj}}{dT},\theta_{2}^{\varepsilon }=\frac{1}{2}\frac{1}{\varepsilon_{kj}}\frac{d^{2}\varepsilon_{kj}}{dT^{2}},\theta_{1}^{\rho }=\frac{1}{\rho }\frac{d\rho }{dT},\theta_{2}^{\rho }=\frac{1}{2}\frac{1}{\rho }\frac{d^{2}\rho }{dT^{2}}\\ \theta_{1}^{\alpha i}=\frac{1}{\alpha i}\frac{d\alpha i}{dT},\theta_{2}^{\alpha i}=\frac{1}{2}\frac{1}{\alpha i}\frac{d^{2}\alpha i}{dT^{2}} \end{array}$$

$\left(\theta_{1}^{C},\;\theta_{2}^{C}),\;(\theta_{1}^{e},\;\theta_{2}^{e}),\;(\theta_{1}^{\varepsilon },\;\theta_{2}^{\varepsilon }),\;(\theta_{1}^{\rho },\;\theta_{2}^{\rho }),\;(\theta_{1}^{\alpha i},\;\theta_{2}^{\alpha i}\right)$ are the first and second order temperature constants of stiffness, piezoelectric, dielectric, density and thermal expansion coefficient, respectively. The direct coupling between temperature variation and strain, stress and electric field is difficult and time-consuming in the simulation process. In the following work, the detailed mechano-thermal coupling analysis of experimental changes was carried out. 

From Equation (1), the resonant frequency is determined by the SAW velocity v of the substrate and period λ of an IDT. Therefore, the deviation of the resonant frequency can be described [28] as

(14) $$df_{0}=-\frac{v}{\lambda^{2}}d\lambda +\frac{1}{\lambda }dv$$

where $f_{0}$ is the initial resonant frequency. The substrate is mainly subjected to strain changes, and the velocity change is sufficiently small to ignore the second term in Equation (12).

(15) $$df_{0}\approx -\frac{v}{\lambda^{2}}d\lambda =-f_{0}\varepsilon_{x}$$

where $\varepsilon_{x}=\frac{d\lambda }{\lambda }$ is the strain.

The change in the resonant frequency can be obtained by extracting $\varepsilon_{x}$ at the position of the SAW pressure or vibration sensor. It is assumed that the stress and strain are not much different in the coverage area of the SAW resonator. The values at the center point of the SAW resonator were selected to represent all areas of the resonator to simplify the model.

The pressure sensitivity $K_{fp}$ of the pressure sensor is defined as

(16) $$K_{fp}=\frac{\Delta f}{f_{0}}\frac{1}{P}=-\varepsilon_{x}\frac{1}{P}$$

where *P* is the pressure, $f_{0}$ is the reference frequency, and $\Delta f=f-f_{0}$ is the frequency change.

The acceleration sensitivity of the vibration sensor is defined as

(17) $$K_{fa}=\frac{\Delta f}{f_{0}}\frac{1}{\alpha }=-\varepsilon_{x}\frac{1}{a}$$

where a is the acceleration.

The temperature properties of the substrate play a vital role in improving the performance of SAW temperature sensors. However, in pressure and vibration sensors, it is necessary to minimize the interference of temperature effects on the sensors. When the temperature changes, the elastic constant $C^{E}$, piezoelectric constant e, dielectric constant $\varepsilon^{S}$, density *ρ* of the substrate material change accordingly [29]. Without loss of generality, set the material constant X(T), and expand its Taylor $T_{0}$.

(18) $$\begin{array}{l} X(T)=X(T_{0})[1+\frac{1}{X(T_{0})}\frac{dX(T)}{dT}|{}_{T=T_{0}}(T-T_{0})+\\ \frac{1}{2X(T_{0})}\frac{d^{2}X(T)}{dT^{2}}|{}_{T-T_{0}}(T-T_{0})+\ldots ] \end{array}$$

where $\frac{1}{X(T_{0})}\frac{dX(T)}{dT}$ and $\frac{1}{2X(T_{0})}\frac{d^{2}X(T)}{dT^{2}}$ are the first and second order temperatures material coefficient, respectively. When the temperature change is relatively small, the first two items are retained in the formula.

**Figure 1:**
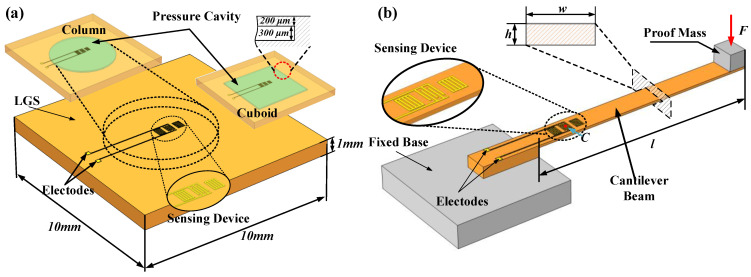
Diagram of the (**a**) pressure sensing device and (**b**) vibration sensing device.

As shown in Figure 1, the pressure and vibration sensors analyzed in this paper adopt LGS as the base material and adopt a single-port resonator. According to Equation (12), the response of an SAW resonator is proportional to the strain where it is located. The displacement and stress of the substrate reflect the degree of structural deformation to avoid damage to sensitive structures. So the stress, strain and displacement distribution of the SAW pressure sensor with column and cuboid cavities were analyzed numerically. The frequency responses of the SAW sensors placed at the edge and center of the cavity are compared. For a pressure sensor, the substrate is a square crystal of 10 mm × 10 mm × 1 mm, and a cylindrical sealed cavity with a diameter of 6 mm is set in the central area of the substrate. A rectangular sealed cavity of 6 mm × 6 mm was also simulated to compare with the cylindrical sealed cavity. The height of the cavity is 300 μm, and the top of the cavity is a LGS crystal film with a thickness of 200 μm. The vibration sensor adopts a cantilever beam *l* mm × *h* mm × *w* mm made by LGS as the sensitive structure with one end fixed on the base. A mass is fixed on the other undamped end as a load to improve the performance of the vibration sensor.

## 3. Analysis of Simulation Results

### 3.1. Temperature Sensor Simulation

**Figure 2:**
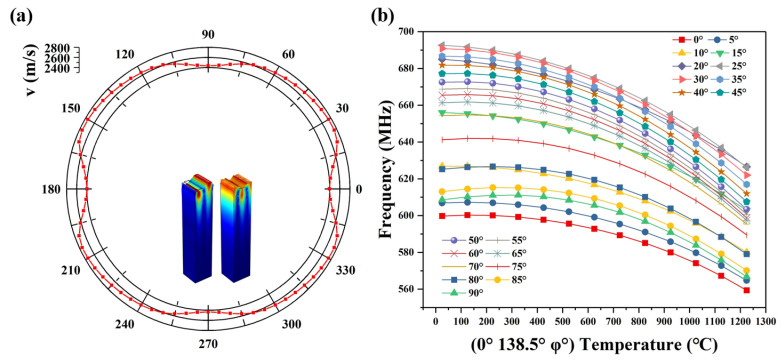
Properties of the LGS substrate. (**a**) Acoustic velocity distribution. (**b**) Temperature response of SAW sensors with λ = 4 μm along different propagation directions φ.

In this paper, a SAW temperature sensor is manufactured with the LGS substrate, and temperature sensitive tangential simulation analysis is carried out on the LGS substrate. Table 1 describes LGS parameters. COMSOL simulation software was used for simulation, and related parameters were set in Table 1. The most sensitive tangential angle of the LGS substrate in tangential direction (0, 138.5, φ), (φ∈[0, 360]) was analyzed. The article simulates a sensor with a finger width of 1 μm, and the specific parameters are shown in Table 2. Simulation results are shown in Figure 2a. It follows that LGS has symmetry. The calculated period of the (IDTs) of the temperature sensor is 4 μm with platinum as the electrode. The phase velocity of the (0°, 138.5°, φ°) LGS is between 2400~2800 m/s, and reaches the maximum at about φ = 25° (Figure 2a). According to the results in Figure 2a, we can see that the range of 0–90° is selected to simulate and analyze the relationship between temperature and frequency within the range of 0–1200 °C. As can be seen from the Figure 2b, the temperature-frequency curve has obvious parabolic characteristics. Temperature-frequency curves such as φ = 80° and 90° show turnover points which are not appropriate for sensing. The simulation results show good agreement with the experimental data [20]. The (0°, 138.5°, 25°) LGS is suitable for the propagation direction of the SAW temperature sensor in consideration of high phase and high temperature sensitivity.

**Figure 3:**
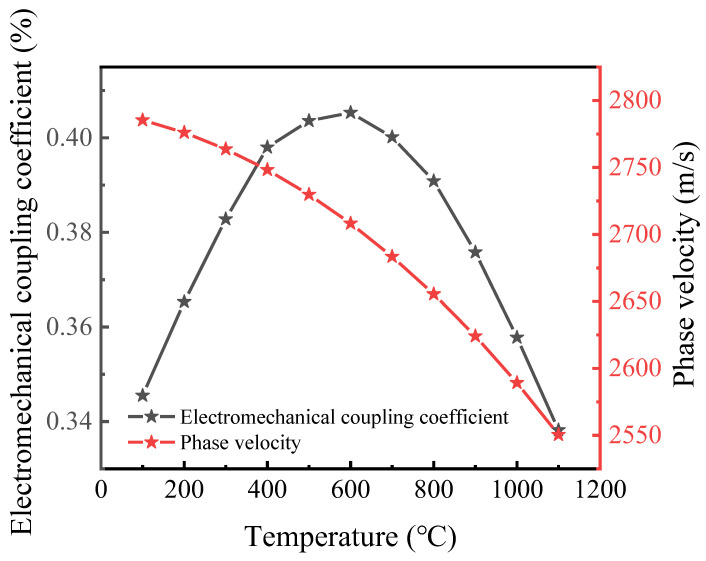
The electromechanical coupling coefficient (K^2^) and phase velocity (v) with different temperature.

Figure 3 describes the electromechanical coupling coefficient (K^2^) and phase velocity (v) variation from 25 °C to 1100 °C. It can be seen from the figure that the phase velocity decreases with the increase of temperature. The mechanical and electrical coupling coefficient shows a quadratic curve with the increase of temperature. At about 600 °C, the K^2^ reaches the maximum, that is, the performance of the surface acoustic wave sensor is the best. With the continuous increase of temperature, the performance of SAW decreases.

### 3.2. Pressure Sensor Simulation

**Figure 4:**
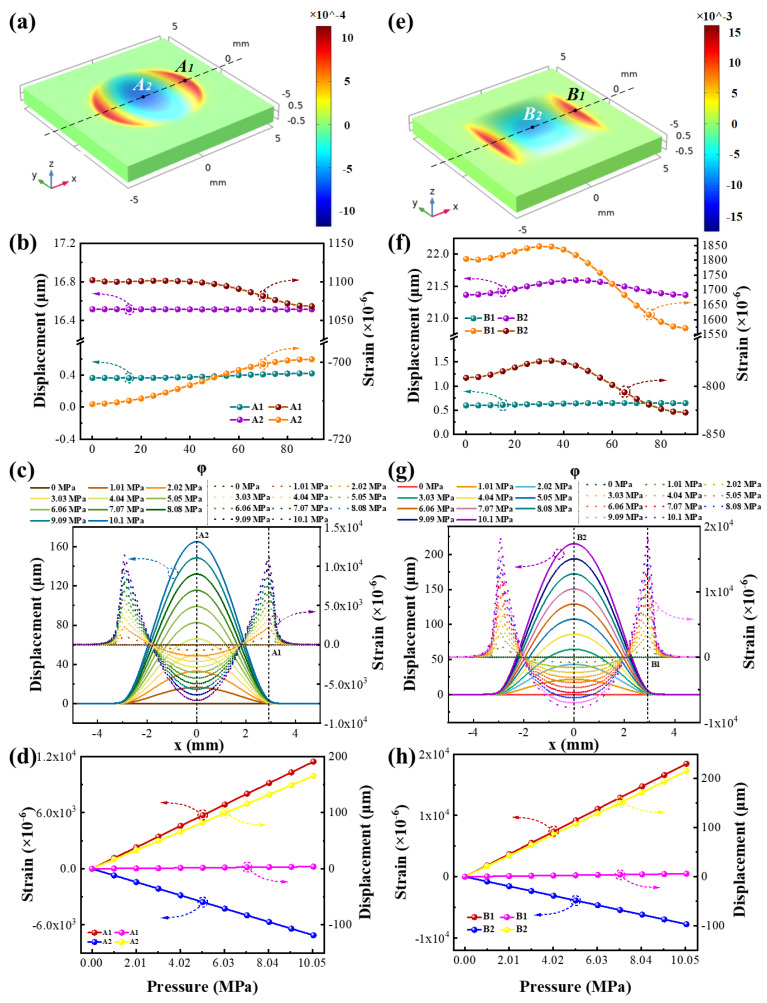
(**a**) Strain distribution in the direction of φ = 0° of column cavity with pressure = 1.01 MPa. (**b**) The relationship between the strain and displacement at A1 and A2 as a function of φ, pressure = 1.01 MPa. (**c**) Curve of strain and displacement of column cavity in the direction of φ = 0° as a function of pressure. (**d**) The displacement and strain (φ = 0°) of A1 and A2 under different pressures. (**e**) Strain distribution in the direction of φ = 0° of cuboid cavity with pressure = 1.01 MPa. (**f**) The relationship between the strain and displacement at B1 and B2 as a function of φ, pressure = 1.01 MPa. (**g**) Curve of strain and displacement of cuboid cavity in the direction of φ = 0° as a function of pressure. (**h**)The displacement and strain (φ = 30°) of B1 and B2 under different pressures.

Figure 4a shows the strain distribution in the x direction of the column sealed cavity at a pressure of 1.01 MPa. According to Equation (13), the pressure response of the SAW resonator depends mainly on the strain where the sensor is located, so the propagation direction of the resonator is set to the x direction (geometric coordinates). By changing the material coordinate system of LGS, the strain where the sensor is located versus different φ can be obtained. As can be seen from Figure 4a, when the cylindrical cavity is deformed under pressure, the strain at the edge of the cavity surface is positive (red), which means that it has a negative pressure response according to Equation (13). The uniformity of the lateral strain can change drastically due to the semi-moon shape of this region, which leads to the sidelobe effect of the SAW resonator. In contrast, the strain in the center region is negative (blue), and the resonator response in this region has a positive trend. Figure 4b is the displacement and strain of A1 and A2 versus φ when the pressure is 1.01 Mpa. A1 is the maximum strain in the x direction of the surface of the cylindrical cavity, and A2 is the center of the surface of the cylindrical cavity. The displacement at point A1 is much smaller than the displacement at A2 under the same pressure, and the change of displacement at two points versus φ can be ignored. However, the strains at A1 and A2 both reached the maximum when φ = 0°, which were 1101.88 × 10^−6^ and −710.87 × 10^−6^, respectively. Therefore, the edge area of the cylindrical cavity is more suitable for the setting of the resonator compared with the center area. Figure 4c shows the displacement and strain distribution on the surface of the circular cavity along the x direction under different pressures (φ = 0°). It can be seen from the figure that the strain variation at point A2 (central area) is relatively smoother than the strain variation at the edge of the cavity. If the strain variation in the area covered by the resonator pattern is not uniform, the response of the resonator is chaotic. Therefore, it is more appropriate to choose the position of the resonator in the center of the cavity from the perspective of suppressing the resonator interference. Figure 4d shows the displacement and strain (φ = 0°) of A1 and A2 under different pressures. It can be seen that the displacement and strain at A1 and A2 have a linear relationship with the pressure change, and the sensitivity are −1101.88 ppm/MPa and 710.87 ppm/MPa, respectively. Figure 4e shows the strain distribution in the x direction of the rectangular sealed cavity at a pressure of 1.01 MPa. From Figure 4e, it can be seen that when the rectangular cavity is deformed under pressure, the strain at the edge of the cavity is more uniformly distributed in the lateral direction, which means that the SAW resonator at this position has a smaller lateral influence on the sensor. In contrast, the strain distribution in the central region of a rectangular cavity is similar to a rectangle, and has a better consistency in the transverse and longitudinal directions than the oval strain distribution in the central region of a cylindrical cavity. Figure 4f is the displacement and strain of B1 and B2 versus φ with the pressure of 1.01 MPa. B1 is the maximum strain on the surface of the rectangular cavity in the x direction, and B2 is the center of the surface of the rectangular cavity. The displacement at point B1 is much smaller than the displacement at B2 under the same pressure. The displacement at point B2 changes with φ and reaches the maximum when φ = 45°. In contrast, the displacement at point A2 (the center area) is hardly affected by φ. This is mainly because the cross-section of the cylindrical cavity is circular and geometrically isotropic. However, the rectangular cavity causes the displacement to be symmetrically distributed from φ = 0–45° and φ = 45–90°, but the displacement in the range of 0–45° is anisotropic. The strains at points B1 and B2 reach the maximum at φ = 30° and φ = 90°, which are 1847.13 × 10^−6^ and −827.48 × 10^−6^, respectively. Figure 4g shows the displacement and strain distribution on the surface of the rectangular cavity along the x direction under different pressures (φ = 0°). It can be seen from the figure that the strain variation at point B2(central area) is relatively smoother than the strain variation at the edge of the cavity. Compared with the strain distribution of the edge and center of the circular cavity, the strain of the edge and the center of the rectangular cavity is larger, and the strain distribution of the center of the rectangular cavity is smoother and more uniform, which makes it more suitable for sensors. Figure 4h shows the displacement and strain of B1 and B2 under different pressures (φ = 30°). It can be seen that the displacement and strain at B1 and B2 have a linear relationship with the pressure change, and their sensitivity is −1847.13 ppm/MPa and 827.48 ppm/MPa, respectively.

**Figure 5:**
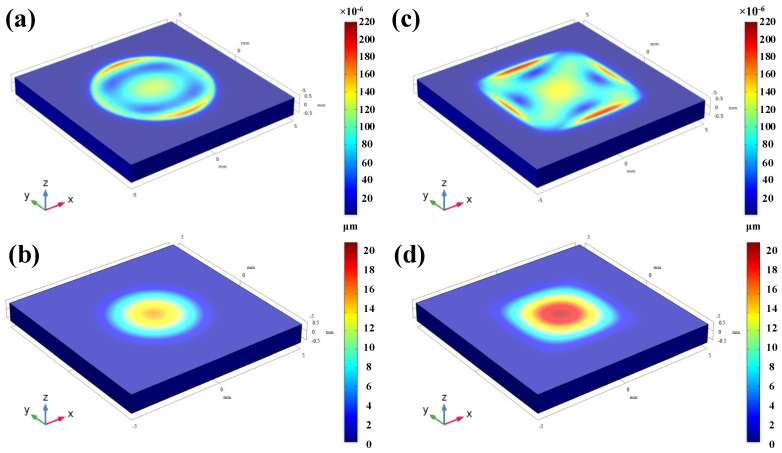
(**a**) Pressure = 1.01 MPa φ=0° Von Mises Stress; (**b**) Pressure = 1.01 MPa φ=0° Total Displacement; (**c**) Pressure = 1.01 Mpa φ=30° Von Mises Stress; (**d**) Pressure = 1.01 MPa φ=30° Total Displacement.

Figure 5a,c are the stress distribution of circular and rectangular sealed cavities at 1.01 MPa, respectively. It can be seen that the stress distribution of the circular sealed cavity has anisotropy, which is the same in the rectangular sealed cavity. The stresses at the edges of the circular and rectangular sealed cavities are greater than the stresses in the central region, which means that cracking of the LGS film is more likely to occur in the edge region. Figure 5b,d are the displacement distribution diagrams of circular and rectangular sealed cavities at 1.01 MPa. Under the same pressure, the maximum displacement of the circular seal cavity is greater than the maximum displacement of the rectangular seal cavity, and the displacement of the circular seal cavity is in a concentric circular distribution. Compared with the displacement of the circular sealed cavity, the displacement of the rectangular sealed cavity is smoother and more balanced, so the circular cavity is more likely to rupture.

### 3.3. Vibration Sensor Simulation

**Figure 6:**
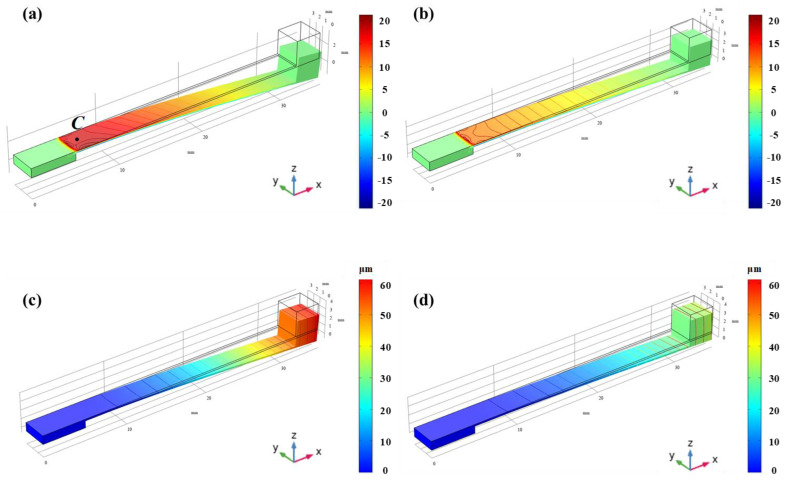
Von mises stress and total displacement profiles of LGS cantilever beam in different propagation directions with an acceleration of 1 g. (**a**) Acceleration = 1 g φ=35° Von Mises Stress (**b**) Acceleration = 1 g φ=35° Total Displacement (**c**) Acceleration = 1 g φ=90° Von Mises Stress (**d**) Acceleration = 1 g φ=90° Total Displacement.

Figure 6a shows the strain distribution along the x-direction for an LGS cantilever beam with φ = 35° at an acceleration of 1 g. It can be seen that the strain near the fixed end region is greater than that in the undamped end region, which means that the SAW resonator located in this region has better performance. Figure 6b shows the strain distribution along the x-direction for an LGS cantilever beam with φ = 90° at an acceleration of 1 g, and its maximum strain is 12.4 × 10^−6^. However, the maximum strain is 21.4 × 10^−6^ with φ = 35°. Figure 6c,d are the displacement distributions of the cantilever beam when φ = 35° and φ = 90°, respectively. The maximum displacement of a cantilever beam with φ = 35° is greater than the maximum displacement of a cantilever beam with φ = 90°. The greater the strain or the greater the displacement, the greater the risk of cantilever beam fracture. Therefore, while improving the performance of the sensor, the risk of fracture of the cantilever beam must be weighed.

**Figure 7:**
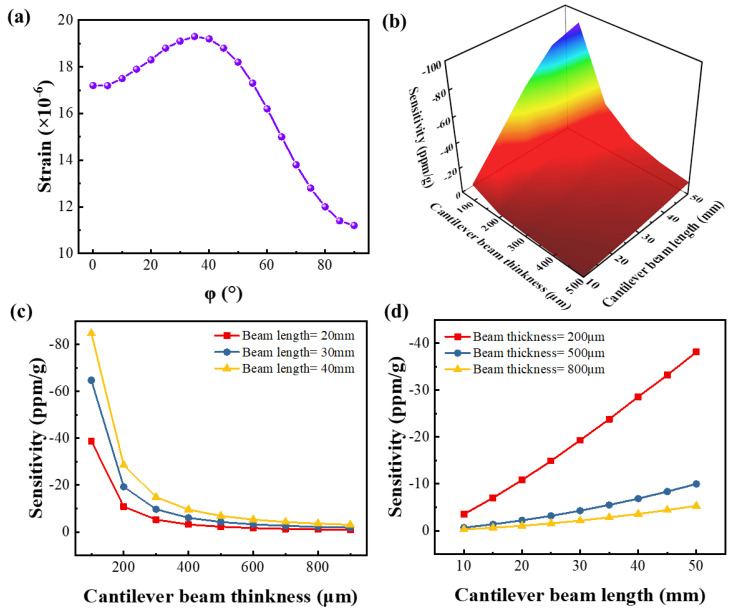
(**a**) Acceleration sensitivity in different directions of propagation of (0°, 138.5°, φ°) LGS. (**b**) The calculated relationship among the sensor sensitivity and the cantilever beam length and thickness (φ = 35°). (**c**) The calculated relationship among the sensor sensitivity and the cantilever beam thickness with beam length of 20, 30 and 40 mm (φ = 35°). (**d**) The calculated relationship among the sensor sensitivity and the cantilever beam length with beam thickness of 200, 500 and 800 μm (φ = 35°).

Figure 7a is the curve of the strain along the x-direction at point C as a function of the φ. As can be seen from the figure, the strain of the cantilever beam is the largest at φ = 35°, which is 19.3 × 10^−6^, and the strains in different tangential directions are all positive values, so the resonator at this point has a negative frequency response. Figure 7b shows the relationship between the sensitivity of the resonator at point C and the length and thickness of the cantilever beam. When the cantilever beam is deformed under acceleration, its lateral strain is almost negligible, and it has little effect on the frequency of the resonator, so we ignore the effect of the width of the cantilever. Figure 7c shows the vibration sensitivity of the resonator at point C of the cantilever beam with different thicknesses when the cantilever length is 20, 30, and 40 mm. When the length is fixed, the sensitivity of the resonator increases with the thickness of the cantilever. The theoretically calculated sensitivity is −84.7 ppm/g (l = 40 mm, t = 100 μm). Figure 7d shows the vibration sensitivity of the resonator at point C of the cantilever with different lengths when the thickness of the cantilever is 200, 500 and 800 μm. When the thickness is fixed, the sensitivity of the resonator increases with the length of the cantilever beam. The theoretically calculated sensitivity is −38.1 ppm/g (l = 50 mm, t = 200 μm). The longer or thinner the cantilever beam, the higher the sensitivity of the resonator. However, an increase in length or a decrease in thickness results in a decrease in the strength of the cantilever beam, leading to the fracture of the cantilever beam. The possibility of such fracture is greater when the cantilever beam resonates.

### 3.4. Simulation Design of Multi-Parameter Integrated Sensor

According to the above simulation analysis, the tangential sensitivity of temperature, pressure, and vibration single parameter sensors are obtained at (0°, 138.5°, 25°), (0°, 138.5°, 30°) (0°, 138.5°, 35°), respectively.

The tangential direction and propagation direction of the Langasite wafer are determined by the Euler Angle [30]. According to the way of determining the Euler angle, it is known that the Langasite crystal has symmetry. Assume that the required index is α, according to the symmetry of LGS crystals [31]:

(19) $$\begin{array}{l} \alpha (\theta +120,\phi ,\psi )=\alpha (\theta ,\phi ,\psi )\\ \alpha (\theta ,-\phi ,\psi )=\alpha (180-\theta ,\phi ,\psi )\\ \alpha (\theta ,\phi ,-\psi )=\alpha (-\theta ,\phi ,\psi ) \end{array}$$

Therefore, it is only necessary to consider the angle study within the range of ϕ=−60°∼60°,ϕ=0°∼180°, ψ=0°∼180°, without considering the entire Angle space between 0 and 360° for each Euler Angle. 

**Figure 8:**
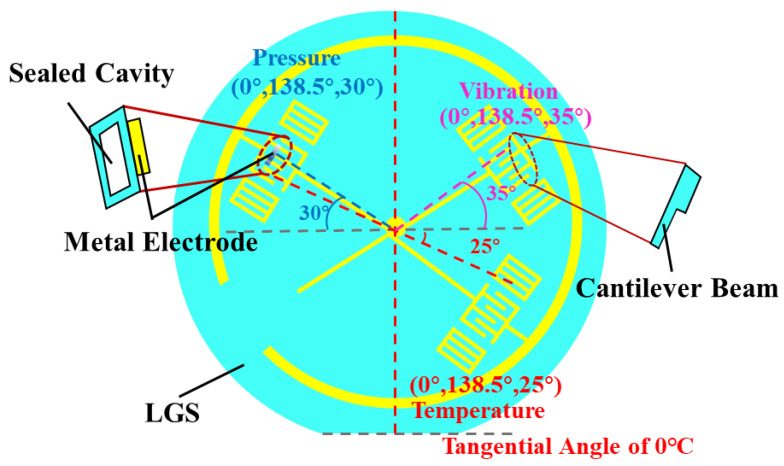
Multi-parameter coplanar layout.

In order to design the multi parameter coplanar integrated sensor, the reasonable layout of multi resonators should be considered. According to the symmetry of LGS wafer, the reasonable layout of multi resonators can be arranged. The layout diagram is shown in Figure 8.

**Figure 9:**
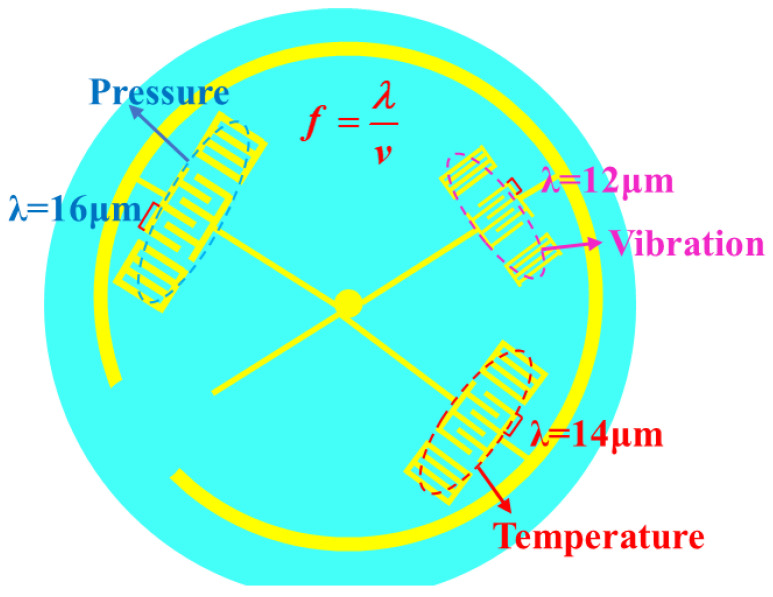
Frequency division design diagram of each parameter.

Multi-parameter coplanar integration makes crosstalk easily occur between the corresponding sensitive frequencies within the full range of each parameter. In order to solve this problem, this paper designs a design method of non-intersection of sensitive frequency intervals in each range. According to the measurement range, the separation rule and model of parameter sensitive frequency are established, and the reasonable width of the interdigit is adjusted to make the sensitive frequency interval in each range have no intersection. The design is shown in Figure 9.

**Figure 10:**
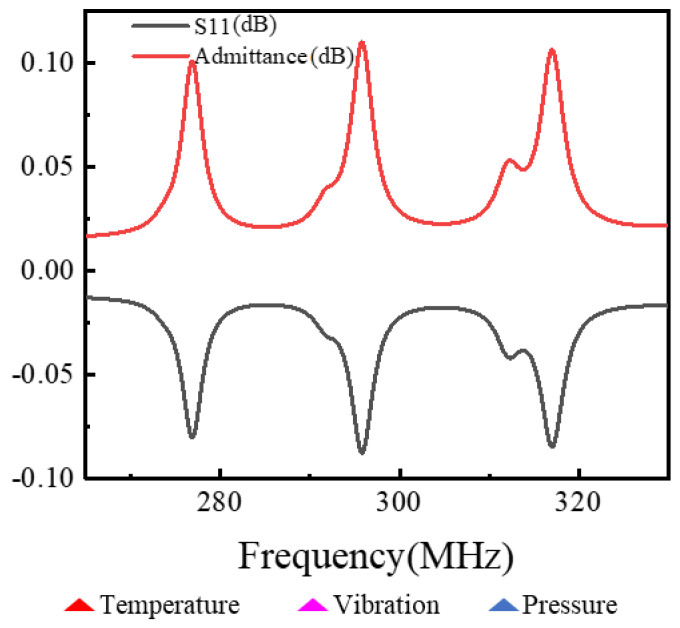
Three-parameter simulation design drawing.

According to the above design rules, the three-parameter coplanar integrated sensor is simulated, and the simulation results are shown in Figure 10. As can be seen from Figure 10, multi-parameter frequency division can be realized by using the above design idea, which provides ideas for the manufacturing of multi-parameter coplanar integrated sensors. Julius Koskela’s team [22] used lithium niobate as the base for simulation and simulated the admission curve, and the response result was two orders of magnitude lower than the simulation result in this paper.

## 4. Conclusions

In this paper, the optimized structures and sensitive orientations of temperature, pressure, and vibration sensors based on (0°, 138.5°, φ°) LGS were obtained through numerical simulation. The results showed that (0°, 138.5°, 25°) is the suitable cut for high phase velocity and high temperature sensitivity without a temperature turning point. For pressure, the edge position of a square cavity is recommended owing to its stress uniformity and high sensitivity. At this position, (0°, 138.5°, 30°) is the optimal cut. For the vibration sensor, (0°, 138.5°, 35°) is the optimal cut, and the optimized length and thickness of the cantilever beam are determined according to the analysis. The single-parameter simulation conclusion lays a theoretical foundation for the simulation of multi-parameter integrated sensors for future work. In this paper, the optimal sensitive tangential direction of each parameter and the crosstalk of the corresponding sensitive frequencies within the full range of each parameter are analyzed, determining the integrated multi-parameter distribution and more sensitive parameters for each frequency separation rule and coplanar models. It provides a method and theoretical basis for sensor manufacturing and a way of thinking to meet the requirement of simultaneous measurement of multiple parameters under harsh environments.

## Figures and Tables

**Table 1:** The value of the acoustical physical constants and their temperature coefficient of LGS.

Material Constants		Absolute Quantities	Temperature Coefficients 1st 10^−6^/K	Temperature Coefficients 2nd 10^−9^/K
Elastic constants [×10^9^, N/m^2^]	C11^E^	189	−58.4	−82.7
	C12^E^	104	−98.7	−51.9
	C13^E^	102	−82.3	−68.1
	C14^E^	14.40	−307.0	95.5
	C33^E^	268	−105.0	−89.7
	C44^E^	53.3	−58.7	−103.6
Piezoelectric constant [C/m^2^]	e11	−0.438	464.5	−427.5
	e14	0.104	−700.3	1600
Dielectric constants	ε11/ε0	19.06	135.5	118.0
	ε33/ε0	51.60	−783.4	661.2
Coefficient of thermal expansion [ppm/K]	α11	5.08	-	-
	α33	3.49	-	-
Density [kg/m^3^]	ρ	5743	−15.71	-

**Table 2:** The parameters of the resonators of LGS.

Parameters	Values
SAW wavelength (λ, μm)	4
Width of IDT (μm)	1
Pt Electrode thickness (nm)	150
Metal ratio	0.5
Aperture	50 λ
TCF (ppm/k)	−15.8

## Data Availability

Data sharing is not applicable to this article as no new data were created or analyzed in this study.
